# Polymer Amide as an Early Topology

**DOI:** 10.1371/journal.pone.0103036

**Published:** 2014-07-21

**Authors:** Julie E. M. McGeoch, Malcolm W. McGeoch

**Affiliations:** 1 Department of Molecular and Cellular Biology, Harvard University, Cambridge, Massachusetts, United States of America; 2 PLEX LLC, Fall River, Massachusetts, United States of America; University of Akron, United States of America

## Abstract

Hydrophobic polymer amide (HPA) could have been one of the first normal density materials to accrete in space. We present *ab initio* calculations of the energetics of amino acid polymerization via gas phase collisions. The initial hydrogen-bonded di-peptide is sufficiently stable to proceed in many cases via a transition state into a di-peptide with an associated bound water molecule of condensation. The energetics of polymerization are only favorable when the water remains bound. Further polymerization leads to a hydrophobic surface that is phase-separated from, but hydrogen bonded to, a small bulk water complex. The kinetics of the collision and subsequent polymerization are discussed for the low-density conditions of a molecular cloud. This polymer in the gas phase has the properties to make a topology, viz. hydrophobicity allowing phase separation from bulk water, capability to withstand large temperature ranges, versatility of form and charge separation. Its flexible tetrahedral carbon atoms that alternate with more rigid amide groups allow it to deform and reform in hazardous conditions and its density of hydrogen bonds provides adhesion that would support accretion to it of silicon and metal elements to form a stellar dust material.

## Introduction

Hydrophobic polymer amide (HPA) analyzed by electron microscopy and electron diffraction, has been shown to form an encapsulating skin over water [1]. The hydrophobic polymer was attached to, but separate from, a face of bulk water, the whole with hydrogen bonding from water to water and polymer to water. Entrapment of water in that work was also dependent on the fundamental property of polymer amide [2], [3] of flexible tetrahedral carbon atoms alternating with the more rigid amide groups allowing curvature. In this paper we consider the possibility that this material, which is represented in living organisms by a protein that has remained essentially unchanged in its DNA code sequence for 3.8 Gy on Earth, could have formed as a type much earlier in the universe when its constituent elements first came into existence. We discuss below the astrophysical data that shows 12.7 Gy before the present (12.7 Gya) to be the earliest time for H, C, N and O to co-exist in a density and temperature range suitable for gas phase reactions to lead to amino acids, the latter being the basic units of HPA. We then examine the gas phase reactions of amino acids, taking terrestrial types as a first case in point, in order to find whether gas phase polymerization is likely, and to determine the fate of the water released in the amino acid polymerization (condensation) reaction. We find that water of condensation tends to collect on one side of the polymer thus starting the bulking process of water.

### 1.1 Time line for H,C,N,O chemistry: formation of elements

**Figure 1:**
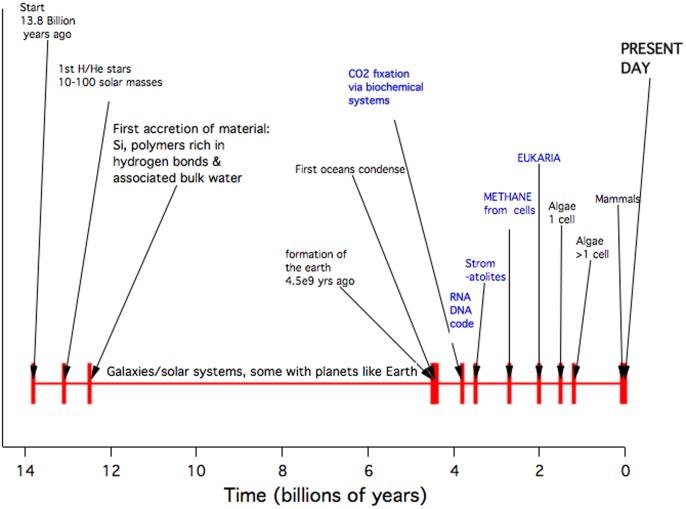
Chemical Time Line.

The current standard model of cosmology based on the Wilkinson Microwave Anisotropy probe (WMAP) sets the age of the Universe at 13.772±0.059 billion years [4], [5]. The era from then to now is displayed as a chemical time line (Fig. 1), with the latter part of the time showing the chemical systems dependent on nucleotide-code-based synthesis on Earth. Here we are concerned with earlier chemical systems that arose following the cataclysmic production of large quantities of oxygen, nitrogen and carbon in the era of the first stars, prior to 12.7 Gya.

In the primordial nucleo-synthesis [6] the isotopes of hydrogen and helium, together with a small amount of lithium were produced. Later in the expansion of the universe (at roughly 400,000 years) the free electrons re-combined with these nuclei to produce neutral atoms of H, D, ^3^He, ^4^He and ^7^Li [7]. Accretion of first generation stars from essentially H and He was aided by dark matter “haloes” in which baryonic matter became gravitationally concentrated, then collapsed into massive stars, particularly in the range 63 to 130 solar masses [8], [9]. The predicted end-product of nucleo-synthesis in these “population III” stars was a stellar core comprising mostly oxygen, with some carbon and lesser amounts of nitrogen and higher mass elements. The life of these stars was short and within them terminal He burning lead to collapse during a brief period of “pair production” followed by explosive oxygen burning reactions that blew the star apart, or at least caused it to shed much of its mass. The related stellar ultraviolet radiation re-ionized the inter-galactic medium at redshift Z = 11 [10], [11] and by redshift Z = 6 (at 0.9 Gy) large galaxies were prolifically giving birth to stars [12]. The conditions that could lead to polymer amide production therefore first began to exist about 1 Gy into the life of the universe once the necessary elements H, C, N and O were dispersed, the radiation and baryon temperatures had fallen, and the next generation of smaller stars had begun to condense. Locally the density would be raised to at least that of the warm, dense clouds observed in our galaxy, approximately 10^7^ – 10^8^ H_2_ molecules cm^−3^ in a cloud interior equilibrium radiation environment of 100 K or more.

### 1.2 From H,C,N,O to amino acids in space

Our thesis in the present paper is that a polymer of amino acids (HPA) could have formed in gas phase chemistry as the next stage of chemical evolution after formation of the numerous smaller molecules that have been catalogued [13]. If correct, HPA would comprise the first solid density material and the properties of HPA, particularly its richness in hydrogen bond sites, would provide adhesion to start the accretion of other free molecules, also of the metal ions present at low density in early times. Although the simplest amino acid glycine has not been definitively observed in space [13]–[15] its gas phase production is expected. For our proposal to be valid, before interstellar “dust” or “ice” exists to provide the solid density route to glycine [16], there has to be a purely gas phase route from simple molecules to glycine and other amino acids at the relatively low density and temperature of a warm dense cloud. In the laboratory amino acids may be able to form purely in the gas phase when appropriate gas mixtures are subject to electric discharge [17] or ionizing radiation [18]. However, it seems that none of these demonstrations has been performed in conditions where contact with a surface was impossible during the duration of the experiment and therefore an element of surface or volume solid density reaction chemistry could not be completely excluded. In [17], for example, a water phase reaction appeared to account for all of the amino acid synthesis and in [18], a water surface was present. Moreover, the thermal energy and density available in these experiments would allow some reactions to proceed that would not occur at the lower temperatures of interstellar space. A more definitive result obtains when likely individual reactions in the chain are studied in a helium-buffered flow tube [19], in which the reaction rate is high enough to ensure that no contact with a surface occurs. The difficulty with this complementary approach, however, is that very many reactions, possibly numbering in the thousands, have to be studied in order to establish a most probable gas phase reaction sequence to amino acids. For example, a promising gas phase reaction [19] to produce (ionized) glycine, that of ionized hydroxylamine with acetic acid depends upon the synthesis of hydroxylamine which to date is only known to be possible in the solid or liquid state.

In theoretical work toward a gas phase formation route Maeda and Ohno [20] have searched the potential energy surface of glycine to identify a neutral chemistry reaction to glycine via ammonium ylide CH_2_NH_3_, an energetic isomer of methylamine CH_3_NH_2_, in reaction with CO_2_. As ammonium ylide is in principle available via the dissociative recombination of protonated methylamine [20], and the latter may form via the radiative association of NH_3_ and CH_3_^+^ [21], [22], the elements of a truly gas phase route to glycine (and other amino acids) are becoming visible. Glycine has not been detected astronomically, but in view of the reactions we describe below, it would dimerize and pass into HPA fairly rapidly along with other amino acids, reducing the chance of its observation as a free molecule.

### 1.3 Gas phase polymerization of amino acids: background

We address the gas phase reactions of amino acids via *ab-initio* modeling and determine that peptide bonds can form for many amino acid pairs in warm dense cloud conditions, simply following a gas phase collision of two amino acids. The water molecule that is ejected during peptide bond formation remains bound to the nascent di-peptide, and this small molecular complex can grow by accretion, adding additional amino acids or small polar molecules, particularly water.

Our reference point for the study of early HPA was a study of hydrophobic polymer amide of biological origin [1] in which we observed entrapment and ordering of water in vesicles and tubes ranging from nanometers to microns, with movement of these structures demonstrated from 233–298 K. The biological polymer is typically 25 nm long as a stretched-out beta sheet of 75–81 amino acids. The National Center for Biotechnology Information (NCBI) web site [23] provides extensive data on this ancient protein. It is physically tough, some forms functioning in ocean vents at 383 K [24]. Biologically it acts as a rotor component in a 5 nm lipid membrane for the synthesis of cellular ATP. When reconstituted in hydrated systems with lipid it will form ion-conducting pores, even when the substrate is silicon [25].

For the *ab initio* calculation of amino acid collisional polymerization we mostly took pairs of amino acids from the ancient sequence rather than random pairs, based on the assumption that a polymer sequence that has existed essentially unchanged on Earth for 3.8 Gyr (Fig. 1) might reasonably be expected to have a prior history, particularly as it is only a factor of three to get to 12.7 Gyr ago. A contrasting polymer is polyethylene glycol, which does not appear to be able to condense in such conditions.

## Computational Methods

**Figure 2:**
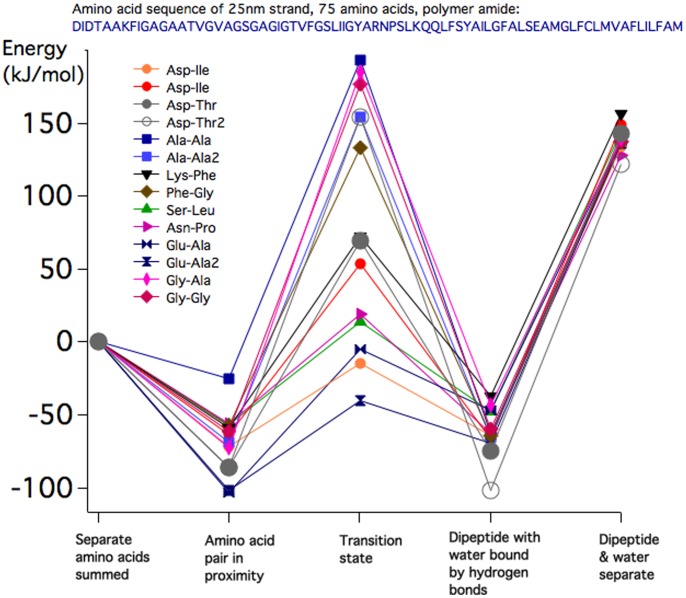
The ordinate depicts the total enthalpy in kJ/mol of 5 molecular states (listed on the abscissa) related to the formation of an amide bond between 14 pairs of gas phase amino acids.

Using Spartan software [26] 14 pairs of amino acids (HPA vertebrate sequence [27]) were manually assembled with the C-terminus of the first <0.4 nm from the N-terminus of the second. The 14 pairs consisted of 10 different pairs with more than one conformer for some pairs and a glycine–glycine pair not in the HPA sequence but included because much prior amide bond work involved just glycine and polymers of glycine. This manual initial molecular geometry was refined prior to any *ab initio* energy calculation by subjecting the pair to energy minimization via molecular mechanics using the MMFF94 force field [28] within Spartan. This usually resulted in an amino acid pair conformation involving at least one intermolecular hydrogen bond connecting the separate amino acids. Then this was followed by *ab initio* total energy equilibrium geometry/transition state geometry density functional calculations: B3LYP/6-31G ([29] Q-CHEM embedded in Spartan) on each of the 5 states involved in the amide bond analysis - depicted on the abscissa of Figure 2. Each amino acid pair was guided through the five states via successive application of MMFF94 followed by more-or-less stationary refinement with B3LYP/6-31G. When we guide the reaction in this manner, the initial conformer determines the remainder of the states. Although the specifics may vary, the energy results do not depend much on the choice of initial conformer, except as discussed in the Results section.

### State 1

Separate amino acid equilibrium energies were computed using *ab initio* total energy/equilibrium geometry density functional calculations (B3LYP/6-31G).

### State 2

The chosen pair of amino acids was manually assembled with the C-terminus of the first <0.4 nm from the N-terminus of the second. This manual initial molecular geometry was refined prior to any *ab initio* calculation by subjecting the pair to a molecular mechanics energy minimization involving the MMFF94 force field. The chosen conformer was then used in an *ab initio* calculation (B3LYP/6-31G) to obtain more accurately the energy of the second of the five states depicted on the abscissa of Figure 2, the amino acid pair in proximity. The graph was adjusted to set the sum of the separate amino acid energies to zero. Other energies are plotted relative to this.

### State 3

A trial transition state in the “trans” configuration was then constructed from the amino acid pair, using Spartan input commands, in a “concerted” configuration, as described by Jensen et al. [30]. We took the amide bond to form via a single “concerted” transition state rather than via a “two-stage” process with two sequential transitions states. In [30] the authors explored these two pathways in detail and found that the concerted and two-stage processes have very similar energy barriers to amide bond formation, with a slightly lower energy to the two-step path. There has not been an experimental determination of the transition state path, to distinguish between these possibilities. Again, starting with the approximate transition state, the B3LYP/6-31G calculation was run to refine the transition state energy and atomic coordinates. These energies are depicted as the third state on the abscissa of Figure 2.

### State 4

Under constraints that preserved the new C-N and O-H bonds that had formed, the system was energy-minimized using MMFF94, to obtain the dipeptide configuration with a water molecule that is usually attached via one or more hydrogen bonds to the dipeptide. Once again the energy and coordinates were refined using the B3LYP/6-31G calculation to obtain the values plotted as state 4 on the abscissa of Figure 2, the dipeptide with attached explicit water.

### State 5

Lastly, separate calculations with B3LYP/6-31G were performed with an isolated water molecule and the isolated dipeptide, to generate the fifth state of Figure 2.

For the calculations on other polymers and poly amino acids the same general approach was taken.

## Results

For a pair of amino acids in state 2, the combined enthalpy of hydrogen bonds is variable between −25 and −100 kJ/mol, and not always the same for two different conformers of the hydrogen-bonded amino acid pair, as seen in the Ala-Ala calculations. An intra-molecular protein hydrogen bond has association energy in the range −12 to −30 kJ/mol, so typically two or more hydrogen bonds are formed between gas phase amino acids in proximity.

The surprising finding was that in nearly half of the di-peptides studied the enthalpy of the transition state, item 3, was lower than or comparable to the separate amino acid enthalpy. The highest energy transition states belonged to the pairs: Ala-Ala (two pairs); Gly-Gly; Ala-Gly and Phe-Gly.

In the fourth item, all bound di-peptide pairs with attached water of condensation showed net binding of about −50 kJ/mol relative to the separate gas phase amino acids. When a water-of-condensation molecule is removed to infinity (the fifth category in Figure 2) an energy input of typically 200 kJ/mol is required, indicating that di-peptide formation in the gas phase is strongly stabilized by the retention of water of condensation.

**Figure 3:**
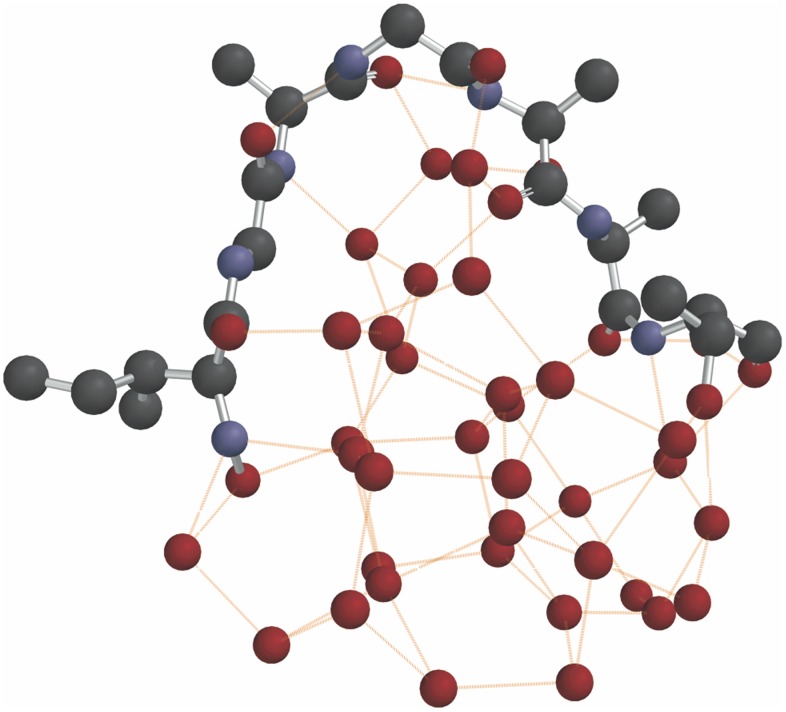
Polymer amide tends to entrap water via intermolecular hydrogen bonding. Starting with 3 water molecules Ile and Gly were added near the water. An amide bond was made between them. In a similar manner further water was sequentially added up to 37 molecules and 5 further amino acids, which were all sequentially amide bonded to the polymer to give Ile-Gly-Ala-Gly-Ala-Ala-Thr. Between any addition, be it water or amino acid, the group was subjected to molecular mechanics MMFF. For clarity the hydrogen atoms are omitted, therefore carbon (back), nitrogen (blue) oxygen (red) and intermolecular hydrogen bonds (orange) only are displayed in figure.

The phase separation of a longer polymer amide from bulking water can only be calculated via molecular mechanics, there being too many atoms for an *ab initio* calculation. Figure 3 shows the energy-minimized conformation of a cluster of 37 water molecules with a 7-mer peptide (Ile, Gly, Ala, Gly, Ala, Ala, Thr) on its surface, using the MMFF94 force field. The model was built in stages with successive energy minimizations, to simulate accretion. The polymer made 15 intermolecular hydrogen bonds to the cluster of 37 water molecules. The water molecules are hydrogen bonded to one another and at the interface with the polymer to the polymer also. This represents a phase separated material topology of hydrophobic polymer on one face and a water cluster on the other. This topological process of phase separation of a water cluster and HPA was observed by us in the experimental work on HPA [1].

**Figure 4:**
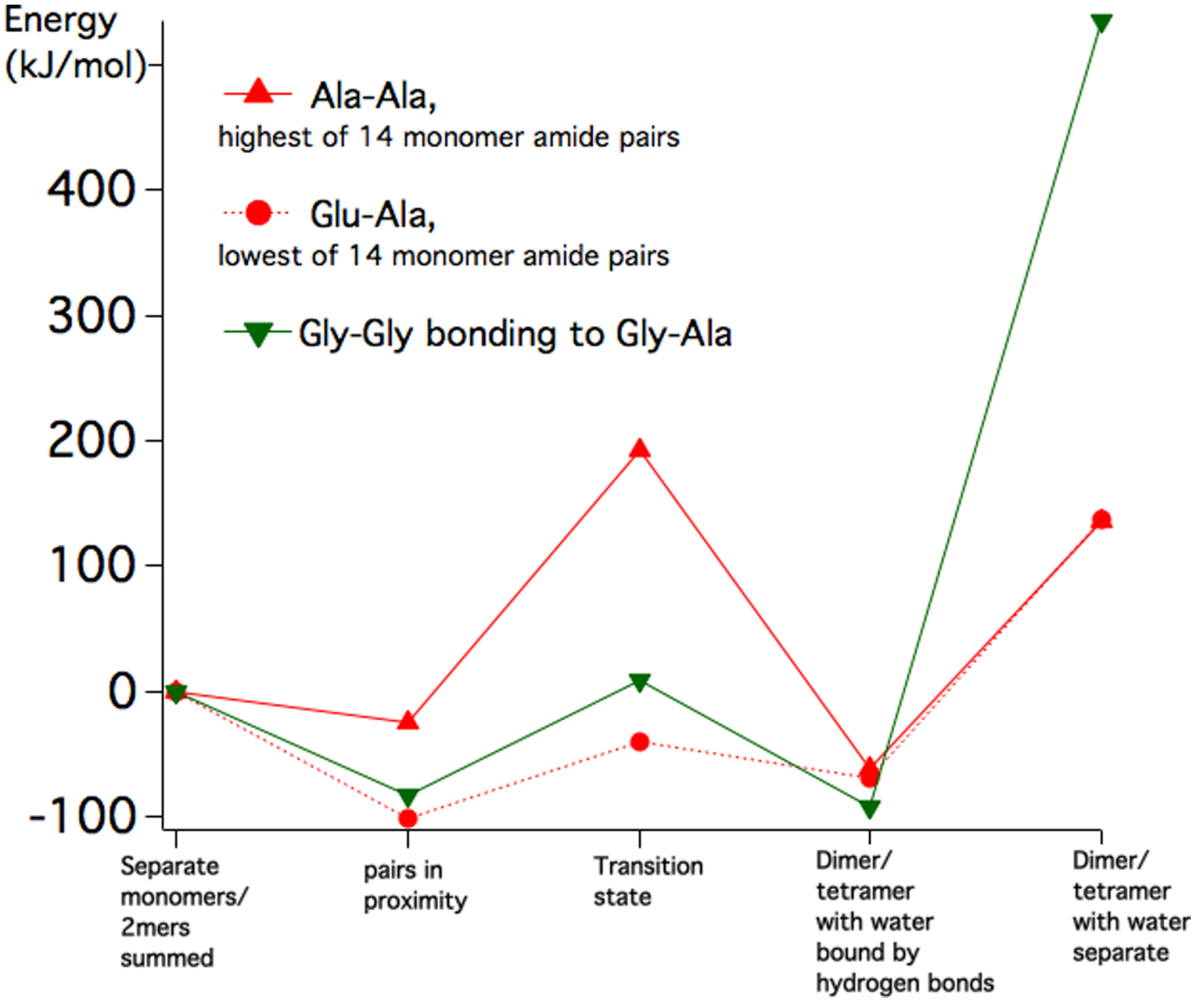
A 4-mer polymer amide formed from 2 dimers (Gly-Gly-Gly-Ala ) has an endothermic energy barrier in the lower range compared to14 separate pairs of amino acids forming dimers.

The high energy barrier for small amino acids like Gly-Gly reduces when a bond is formed between 2 dimers to make a 4-mer. A typical case is shown in Figure 4 for Gly-Gly-Gly-Ala. The initial 2 waters help to attract the dimers to each other by enhancing the polarity of the end groups.

**Figure 5:**
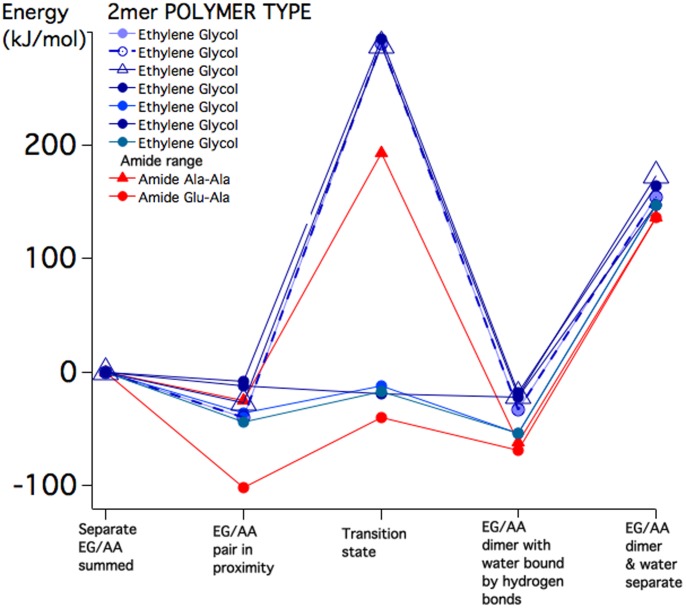
Total energy for 5 states to polymer formation comparing 7 ethylene glycol pairs with the upper and lower range of 14 polymer amide pairs.

The energetics of peptide condensation were compared to those of polyethylene glycol, another candidate polymer for the first material, based on the observation of interstellar ethylene glycol [31]. Figure 5 shows that a pair of ethylene glycol molecules is rather weakly hydrogen bonded (−40 kJ/mol) and that the transition state energy is either high at (290 kJ/mol) or low at zero, in the latter case the bond would not form.

## Discussion

The energy barriers found in the present calculation are similar in magnitude to those calculated [30] for the Gly-Gly pair (200 kJ/mol) and for a model amide bond between formic acid and ammonia (180 kJ/mol), where an equivalent level of ab initio theory was used. In the present work, the use of a wider variety of amino acid pairs begins to uncover the existence in many cases of a much lower barrier to amide bond formation. The results of any one calculation depend upon the initial conformer chosen prior to formation of the transition state. Often, there is not a great difference in outcome for different conformers, but several repeated pairs of amino acids appear in Figure 2 to illustrate the most extreme differences we found. There could be imagined a complete statistical compilation of every amino acid pair in every conformer, followed through every possible transition state with high level theory, but this is a heavy computational task that we hope will be performed in the future.

We found that in general the energy barrier for amide bond formation reduces when an amino acid joins to a pre-existing dipeptide, or higher polymer. The same trend has been noted [32] in regard to poly-glycine in aqueous solution. It is emphasized that the present work only considers non-ionized fully terminated amino acids in vacuo.

The most significant new finding is that amide bond formation can be exothermic when the ejected water molecule remains hydrogen-bonded to the peptide. In the remainder of the discussion we consider the kinetic implications of energetically allowed amino acid condensation in the space environment of warm, dense molecular clouds.

### 4.1 Stability of amino acids and HPA

We compare the life of amino acids in space to the rate of their incorporation into polymer. Amino acids trapped in inert gas matrices at low temperature, simulating the free molecule, have been decomposed by short wave (100 – 200 nm) ultraviolet light from a hydrogen lamp [33]. Depending upon the supposed space environment the half-life of an amino acid to ultraviolet decomposition can vary from 300 yr in a diffuse interstellar medium (DISM) to 3×10^7^ yr in a dark cloud (DC) [33]. Above a certain density, amino acids will collide with each other on a much shorter timescale than this and polymerize into peptides.

The resistance of peptides to short wave ultraviolet light would be expected to be greater than individual amino acids because the energy of a photon absorbed into one bond is communicated throughout many more vibrational modes [34], which equilibrate collisionally, or radiatively [35] with the cool black-body spectrum of the cloud before energy has a chance to re-group and cause dissociation. Even with deep ultraviolet light the destruction efficiency is fairly low. Experimental data on Gly-Trp with 145 nm exposure [36] shows a destruction quantum efficiency of 1.3×10^−2^. Longer wavelength irradiation of Gly-Gly at 206 nm [37] shows a quantum efficiency of 2.2×10^−2^. This data is typically at room temperature, whereas even greater stability against photodecomposition would be expected at lower temperature, especially for longer peptides. Beta sheet peptide structures are very stable in UV light [38].

### 4.2 Polymerization Kinetics

The first stage in amino acid polymerization is the formation of a hydrogen-bonded collision complex (discussed above). In the collision of very small molecules the excess kinetic energy of approach has to rapidly be removed, either radiatively, or by a third body, for the di-molecular complex to be stabilized before the molecules simply fly apart. Larger molecules such as amino acids behave differently upon collision because the kinetic energy of approach is much less than the internal energy and is absorbed via distribution throughout internal rotational and vibrational modes. Consider the collision of molecules containing respectively M and N atoms in a gas at temperature T. The product complex will have 3(M+N−1) internal energy modes and the energy in these modes will equal the initial energy 3(M+N−1)kT/2 plus an increment corresponding to the average kinetic energy donated in the collision, 3 kT/2. The final internal energy per mode will be (M+N)kT/(2(M+N−1)), a relatively small fractional increase. For example, glycine (M = 10) colliding with alanine (N = 13) leads to an effective internal temperature increase by a factor of 23/22 = 1.045. This small excess in temperature does not cause dissociation after the complex has formed. The hydrogen bonding of amino acids in a “collision complex” therefore proceeds as a two-body process. At 100 K the bi-molecular gas kinetic reaction rate (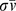) is of the order of 2×10^−11^ cm^3^ sec^−1^ so that, for example, a fractional glycine density of 10^−7^, i.e. 1 cm^−3^, in a warm dense cloud could collide to form a hydrogen-bonded di-peptide complex in 1600 years, which is much shorter than the amino acid ultraviolet decomposition lifetime of 3×10^7^ yr in that environment [33]. The amino acids, once formed, therefore have a high probability of forming the hydrogen-bonded precursor to the dimer.

Moving on to the kinetics of polymerization, Figure 2 shows that the typical binding energy of the hydrogen-bonded di-peptide complex (the amino acid pair in proximity) is 60 kJ/mol (0.6 *eV*), which is shown below to be sufficiently deep to hold the complex together for 1million years at a temperature of 100 K. The excess energy of formation has already been lost on a timescale of 10^4^ seconds by collisions with surrounding thermal He atoms and H_2_ molecules (radiative equilibration with the black body radiation field at the cloud temperature takes two orders of magnitude longer at 100 K). In general the dissociation rate, in the rapid energy exchange (thermalized) limit [35] is given by
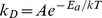
where *k_D_* is the unimolecular dissociation rate constant, *A* is the Arrhenius pre-exponential constant, 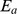
the activation energy of dissociation, *k* Boltzmann’s constant and *T* the temperature. The value of *A* will range from 10^12^ sec^−1^ for “tight” transition states to 10^15^ sec^−1^ for “loose” transition states, as defined in [35].

Hydrogen-bonded dimer complexes form at the rate 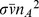 where 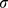 is the collision cross section, 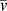 the average particle velocity and 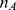 the amino acid number density. If the system reaches equilibrium with respect to this reaction, the ratio of dimer to free amino acid is
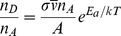
where 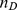 is the dimer number density. As an example, at 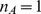 cm^−3^, 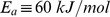 and T = 100 K, the dimer to monomer ratio can range from 3×10^5^ to 3×10^8^ as the pre-exponential coefficient *A* varies from 10^15^ to 10^12^. In these circumstances an amino acid would be overwhelmingly in the di-peptide hydrogen bonded complex.

The final step in polymerization involves passage through the transition state and expulsion of water. With the existence of a stable amino acid pair in proximity the system will progress to form a peptide bond if the transition state energy barrier is less than or of the same order as the typical 60 kJ/mol (0.6 eV) association energy calculated above. Of those studied here, Glu-Ala, Asp-Ile, Ser-Leu and Asn-Pro have transition barriers of less than 0.6 eV (Figure 1), but Lys-Phe, Phe-Gly, Ala-Ala, Gly-Gly and Ala-Gly have considerably higher barriers. In Fig. 2 it is seen that, once formed, the peptide-bonded pairs are stabilized by the continued association of the H_2_O molecule released in bond formation. Although generally the water-stabilized system is at slightly higher energy than that of the initial amino acid pair in proximity, the increased entropy associated with the water will tend to stabilize the peptide bond against dissociation back into the hydrogen-bonded pair.

### 4.3 Formation of tri-peptides and higher polymers

Gas phase condensation of selected amino acids will proceed to tripeptides and higher in typical warm dense clouds. Each water of condensation will stick to the growing molecular system, thereby nucleating the first molecular assemblies of water. At low temperatures such an assembly resembles amorphous ice, with a hydrogen-bonded polypeptide around its surface. To the extent that polar side groups are plentiful, the water will be distributed around the peptide, but more hydrophobic polypeptides will push the water to one side, where it will coalesce with itself (Fig. 3). Other simple molecules such as CO, NH_3_, CH_3_OH and especially H_2_O will collide frequently with the growing complex, finding ready hydrogen bonding to the collective water with the result that interstellar “ice” particles grow in association with the peptide nuclei. The possible evolution in dark interstellar clouds of water-cored peptide-skinned “vesicles” has therefore to be considered. The composition of these first beta sheet peptides will not be random, but will reflect the energetics of the very many pair-wise interactions of interstellar amino acids, many of which will not correspond to those currently present on Earth. Certain combinations will be greatly favored over others on account of the height of the transition state barrier.

### 4.4 Comparison with polyethylene glycol

Another candidate molecule that can hydrogen bond in a di-molecular complex and in principle polymerize producing water of condensation is ethylene glycol. However, we find that the hydrogen-bonded di-ethylene glycol pair has less binding energy than the corresponding amide pair (−40 kJ/mol vs −60 kJ/mol), and this gives dimer to monomer ratios (at 100 K and *n*_EG_ = 1 cm^−3^) ranging between 0.015 and 1.5×10^−5^. Moreover, the transition state barrier into the di-ethylene glycol O-C-C-O-C-C-O structure is 290 kJ/mol (Figure 5), ensuring that negligible amounts of polymer will form, in contrast to the amino acid case. We found that although poly-ethylene glycol (PEG) hydrogen bonds to the surface of a water cluster, it does not link transversely to another PEG molecule, and therefore no skin-forming structure, analogous to the peptide beta sheet, can form.

### 4.5 Outcomes

In summary, beginning approximately 12.7 Gya the Universe, with polymer amide, could have acquired a material that provided a surface and a solid density “platform” to host chemistry in a manner more favorable to many reactions than that of random diffusion in space. The beginnings of polymer amide complexity in this era may have been individual and essentially random, but a trend would be expected to favor polymerization of hydrophobic amino acids on the surface of the water accretion. Even in this era the temperature and available amino acid spectrum would have affected the composition of the polymer formed. Water would have been trapped in bulk form for the first time, in low density conditions where its nucleation would otherwise have been impossible. Such processes would precede poly-aromatic hydrocarbons (PAHs) as possible nuclei for interstellar “dust” [39] because PAH production is only thought to be significant in carbon rich red giant stars, and even there the production rate may not be sufficient to account for the apparent carbon particle accretion rates [40], [41]. Further, polymers involving rings are considered to require a catalyst and a surface to form, and before the first topology arose there was no surface. The composition of the first particles, or “dust” in the early universe is a subject of debate. There is evidence that the spectrum of early ultra-luminous infrared galaxies (ULIRGs) from the period at 1 Gy could be dominated by water emissions, and not mineral dust [42]. This would be consistent with an environment in which gas phase, rather than grain surface chemistry could dominate.

In more recent times accretion around a proto-star could involve HPA of H/C/N/O composition because that type of molecule has a structure to withstand harsh conditions in space and more importantly it is rich in hydrogen bonds for adhesion, the essential actuator of accretion. We are about to start analysis on meteorites that have fallen to Earth having accreted 5 billion years ago when the first elements of our solar system were starting to adhere to one another. HPA if present in such meteorites would not diffuse out based of the fact that rare earth elements in 5 billion years move by less than 700 nm [43]. In ultra-clean slices from the Chelyabinsk and Sutter’s Mill meteorites we will search via focused ion beam and mass spectrometry for polymer using techniques already applied to HPA analysis [1]. Mass spectroscopy has been performed on both meteorites [44], [45] yielding elemental data supportive of H/C/N/O content with amino acids detected in Sutter’s Mill samples. If rugged polymers rich in hydrogen bonds like HPA play an essential role at accretion, we should detect it in these meteorites.

The outer atmosphere of Earth is a “laboratory” environment in which the requisite H,C,N,O reside at low density and temperature. Although amino acids are known to be plentiful in the lower atmosphere [46], there are intriguing questions as to their possible rate of formation and/or polymerization in the upper atmosphere. If existent, this could have implications for water nucleation and precipitation.
